# Tobacco imagery in prime-time television in Spain: A content analysis

**DOI:** 10.18332/tid/204750

**Published:** 2025-06-10

**Authors:** Armando Peruga, Olena Tigova, Ariadna Feliu, Dolors Carnicer-Pont, Laura Anton, Félix Bosch, Juan Miguel Rey-Pino, Esteve Salto, Esteve Fernández, Cristina Martínez

**Affiliations:** 1 Tobacco Control Unit, WHO Collaborating Centre for Tobacco Control, The Catalan Institute of Oncology, Barcelona, Spain; 2 Tobacco Control Research Group, Bellvitge Biomedical Research Institute (IDIBELL), Barcelona, Spain; 3 Centro de Investigación Biomédica en Red of Enfermedades Respiratorias (CIBERES), Madrid, Spain; 4 Center for Epidemiology and Health Policy, Faculty of Medicine, Clínica Alemana Universidad del Desarrollo, Santiago, Chile; 5 Faculty of Medicine and Health Sciences, University of Barcelona, Barcelona, Spain; 6 Department of Primary Care and Public Health, School of Public Health, Imperial College London, London, United Kingdom; 7 Faculty of Medicine, University of Vic – Central University of Catalonia (UVic-UCC), Vic, Spain; 8 Marketing Management and Research Department, University of Granada, Granada, Spain; 9 Department of Health, Government of Catalonia, Barcelona, Spain; 10 Department of Public Health, Mental Health, and Perinatal Nursing, Faculty of Nursing, University of Barcelona, Barcelona, Spain; 11 Philip R. Lee Institute for Health Policy Studies, University of California San Francisco, San Francisco, United States

**Keywords:** public policy, media, advertising and promotion

## Abstract

**INTRODUCTION:**

Exposure to tobacco content in media among youth is a well-established risk factor for smoking initiation and continued use. This study assessed the prevalence and nature of tobacco imagery on Spanish prime-time television (TV) programming and its associations with program characteristics: genre, production nationality, and broadcast timing.

**METHODS:**

A content analysis of 63959 minutes of TV programming in 2021, excluding advertisements and trailers, across 18 broadcast channels examined the presence of tobacco imagery: actual tobacco use, tobacco cultural cues, smoking ban violations, tobacco brand appearances, or any of these.

**RESULTS:**

The analysis revealed that 2.4% of the TV programming time contained at least one instance of tobacco imagery, resulting in 8.5 million impressions for viewers aged 4–24 years. Feature films had the highest prevalence of tobacco-related content (adjusted prevalence ratio, APR=11.9; 95% CI: 9.5–14.9). Tobacco-related content appeared more frequently outside designated children's protection hours (PR=0.7; 95% CI: 0.6–0.80). However, its presence within the designated children's protection schedule remains a significant concern, generating 15.6 million tobacco impressions for young viewers.

**CONCLUSIONS:**

The seemingly modest content level of tobacco imagery (2.4%) translates into a substantial number of impressions for young viewers aged 4–24 years, including during the designated children's protection schedule. Reducing tobacco imagery in films and TV series represents a promising strategy for curbing youth smoking. However, the current reliance on youth protection schedules is inadequate. To better protect children from tobacco imagery, policies should mandate strong anti-tobacco disclaimers preceding programs featuring tobacco and certificates of No Pay-off for tobacco portrayals.

## INTRODUCTION

Exposure to tobacco content in media, particularly among youth, is a well-established risk factor for smoking initiation and continued use^1^. While the prevalence of regular smoking (at least once a month) among participants aged 14–18 years in Spain has decreased from 31.1% in 2000 to 21.0% in 2023, significant concerns remain regarding media influence^2^. Given the pervasive presence of TV sets in Spanish households (99.2% ownership in 2017)^3^, children and adolescents are at high risk of encountering tobacco imagery on this medium. In Spain, children aged 4–13 years watch an average of 2.5 hours of TV daily^4^, making it a powerful vehicle for influencing young audiences’ perceptions and behaviors regarding tobacco.

While research has primarily focused on tobacco imagery in films, it is crucial to understand the extent of exposure on TV to inform effective public health interventions. Existing studies highlight that TV viewing occupies a considerable portion of Spanish children and adolescents’ leisure time, reinforcing the need for further investigation. One study analyzing the top 55 grossing films and highest rated TV series in Spain from 2007 found that both frequently contained tobacco scenes (80% of movies and 75% of TV series). Although films had a higher average number of tobacco scenes per hour (5.8 vs 3.2)^5^, the broader reach of television warrants closer examination. Furthermore, research indicates a concerning trend in top-grossing films from 2005 to 2015: a significant increase in tobacco occurrences and a shift in smoker portrayals from leading to supporting roles^6^, potentially normalizing smoking within popular culture.

These findings highlight the need for further research to comprehensively understand tobacco imagery exposure across the full spectrum of Spanish TV programming, including films.

Relevant Spanish regulations, based primarily on laws enacted in 2005 and strengthened in 2010, mandate a comprehensive ban on tobacco advertising, promotion, and sponsorship as of 2021. This prohibition covers nearly all media formats, including sponsorships, with very limited exceptions mainly restricted to points of sale targeting adult consumers. Specifically for television (including domestic TV, radio, cable, satellite, and information society services), the law bans all forms of tobacco advertising, promotion, and sponsorship. It also prohibits broadcasting programs or images wherein presenters, collaborators, or invited guests are shown smoking, mentioning, or displaying, directly or indirectly, trademarks, trade names, logos, or other symbols associated with tobacco products. Paid placement of tobacco products or brands in media is also banned. Despite these comprehensive measures, understanding the actual prevalence and nature of tobacco imagery remaining on TV is crucial.

To address this gap, the present study aims to measure the prevalence and nature of tobacco content in Spain’s prime-time TV programming in 2021. By providing data on tobacco depictions in TV programming, this study provides a more comprehensive understanding of the potential influence of TV on smoking behaviour in Spain. This information will help develop more effective tobacco control strategies.

## METHODS

### Sample frame

We conducted a content analysis of a sample of TV programs from 18 broadcast channels in Spain. This sample included the 12 general broadcast channels with the largest audience share (Antena3, Telecinco, RTVE1, la Sexta, Cuatro, RTVE2, Factoría de Ficción (FdF), NOVA, ENERGY, 13TV, Divinity, and Neox), three regional channels broadcasting in the local language (TV3 from Catalonia, TVG from Galicia, and ETB1 from the Basque Country), and three channels targeting children (Boing, Clan, and the Disney Channel). Together, these 18 channels accounted for 68% of the audience share, or nearly 95% of the share among free-to-air channels^7^.

We recorded five hours of programming per day from each of the selected channels over seven days across two randomly selected weeks between 26 April and 24 October 2021. The recordings focused on peak viewership times: 8:00 p.m. to 11:59 p.m. (prime time for adults and minors) and 12:00 a.m. to 12:59 a.m. A total of 76500 minutes of programming were recorded.

### Coding procedure

Following the methodology outlined by Lyons et al.^8^, TV programs were segmented into one-minute intervals. Each interval was assessed for at least 10 seconds of actual program content. Intervals containing commercial breaks, including trailers for future programming on the network, were excluded from the analysis. Each minute interval with sufficient program content was then coded for tobacco occurrences, defined as the presentation or representation of a tobacco product or its consumption. These occurrences were classified into five distinct categories^8^:

1. Explicit tobacco use: This includes the visible use of any tobacco or non-pharmacological nicotine product, such as smoking, chewing or sniffing tobacco, and vaping, by a person or character on screen. Each of these behaviors is defined as follows:
  • Smoking: Holding or controlling a lit tobacco product (e.g. a cigarette), regardless of whether the smoke is actively inhaled or exhaled.
  • Chewing tobacco: Placing or having tobacco in the mouth, regardless of whether it is being actively chewed or sucked.
  • Tobacco sniffing: Placing or having tobacco in any nostril, regardless of whether it is actively inhaled through the nose.
  • Vaping: Using or controlling an electronic vaporizer device (heated tobacco products, electronic cigarettes, etc.), regardless of whether the tobacco content is discernible and whether the aerosol is inhaled or exhaled.
2. Implied tobacco use: This includes implied tobacco use conveyed through verbal or non-verbal cues, even if not explicitly shown. Examples include a burning cigarette in an ashtray not under anyone’s control or a smoky atmosphere.
3. Tobacco paraphernalia: This refers to the on-screen presence of unlit or unused tobacco products or related materials, such as tobacco packs, matches, lighters, or ashtrays.
4. Tobacco brand appearance: This involves the unambiguous display of tobacco branding, regardless of whether the brand is sold in Spain. It includes visual branding on tobacco packs, marketing communications within other programs, branded merchandise, and verbal references to such brands.
5. Other references to tobacco: This category covers any verbal or non-verbal reference to tobacco or related products that do not involve actual or implied use. Examples include a song about smoking without any depiction of smoking or a news report discussing the economic performance of a tobacco company.

Tobacco occurrences that spanned two consecutive one-minute intervals were recorded as two distinct occurrences. However, multiple instances of the same category within a one-minute interval were counted as a single occurrence. Occurrences belonging to different coding categories within the same interval were recorded separately. Consequently, each minute interval could have up to five tobacco occurrences, one for each category.

Each one-minute interval was further classified based on its valence as clearly and exclusively positive or negative for health. A tobacco occurrence was deemed negative for health if the observer determined that it portrayed tobacco use, promotion, or the tobacco industry in a positive or neutral light without acknowledging any potential harm to health, the economy, or the environment. On the other hand, positive-for-health occurrences typically included images of no-smoking signs or characters who were diagnosed with, suffering from, or dying from diseases explicitly linked to tobacco use within the storyline. If a one-minute interval contained negative and positive-for-health occurrences, its valence was classified as negative. Occurrences clearly and exclusively positive for health were excluded from the analysis (n=6).

We also coded several program characteristic variables that we hypothesized could be associated with the frequency of tobacco occurrences:

- Program genre: Categorized as entertainment, news, or other. Entertainment programs included animated productions, non-animated feature films and series, reality shows, soap operas, humor programs, and game shows. News programs were defined as those that communicate facts, events, or opinions about them, generally occurring in the present or recent past. These included daily news broadcasts, news magazine shows, debates, press conferences, sports news, weather news, and documentaries. The ‘Other’ category encompassed programs that did not fit into the first two categories, such as purely educational programs and those that cater to specific religious needs.
- Program subgenre: Programs were assigned one subgenre and then grouped for analysis as follows: Daily news (News), other informative programs (documentaries, educational, and other news programs), feature films (animated and non-animated movies made for TV and the big screen), scripted series (except soap operas) and other entertainment programs (contests, humor, soap operas, reality shows, short cartoons, and other entertainment programs).
- Program production nationality: This variable indicates whether the majority share of the production was Spanish, from the USA, or another country. Program information, including genre, subgenre, nationality, and year of production, were determined using program announcements, credits, the Internet Movie Database (https://www.imdb.com/), and channel websites. When this information was unavailable, the researchers relied on their best judgment.
- Channel ownership: We classified the 18 channels as private or government-owned. Private channels belonged primarily to the Mediaset and the A3Media conglomerates. The proprietor of each channel was identified based on information available on the channel’s website.
- Program broadcast timing: Between 8:00 p.m. and 10:00 p.m., broadcasts were considered to occur within a protected time slot for minors. Spanish law establishes such protection from 6:00 a.m. to 10:00 p.m., during which time TV channels are prohibited from broadcasting content deemed unsuitable for viewers under 18 years. This includes programs with graphic violence and sexually explicit scenes. While tobacco-related content is not specifically mentioned as inappropriate for minors, a general provision prohibits programming and advertising that could seriously harm their physical, mental, or moral development or pose a serious risk to public health. Additionally, since 2005 Spanish tobacco control law bans all direct and indirect tobacco advertising and promotion, including on TV^9^.

### Coding reliability

To ensure coding consistency, each observer independently coded every assigned one-minute interval. The research team then reviewed a random 10% sample of all coded intervals. Inter-rater reliability between each observer and the research team’s established criteria for the five types of tobacco occurrences was assessed using Cohen’s kappa statistic^10^. Kappa values ranged from 0.23 (p<0.001) for implicit use to 0.76 (p<0.001) for explicit tobacco use, indicating fair to substantial agreement. Owing to the lower agreement for implicit use, the research team subsequently reviewed and recoded all instances initially categorized as such.

### Analysis

This study analyzed the frequency and types of tobacco occurrences in Spanish TV programming. To facilitate our analysis, we created four outcome variables. First, we combined instances of on-screen explicit and implicit tobacco use into a single category: ‘actual use’. Second, we categorized the presence of paraphernalia and indirect references as tobacco ‘cultural cues’ because they remind the audience of broader cultural norms and attitudes toward tobacco. Third, we created a variable to denote explicit smoking in areas where it is prohibited by Spanish law, termed ‘smoke-free legislation violation’. Finally, we created an overarching category encompassing any tobacco occurrence, defined as any one-minute interval featuring at least one of the five types of coded tobacco depictions. Due to the low frequency of tobacco branding occurrences (n=8), this specific depiction was excluded from further separate analyses.

As independent variables, the analysis considered the following program and channel characteristics: network ownership (national and regional public, as well as private ownership by Mediaset, A3Media, or other private networks), production nationality, broadcast time (during or after the protected time slot for minors), program genre (entertainment or news), and program subgenre (feature films, scripted series, other entertainment [e.g. reality shows, soap operas, humor, and variety programs], daily news, and other informative programs). Given that children’s channels primarily broadcast animated and non-animated feature films and series, the analysis was conducted separately for the 15 general channels and, when relevant, for the three children’s channels.

Unadjusted prevalence ratios (PRs) and their 95% confidence intervals (CIs) were calculated using binomial logistic regression with robust variance. Given that the frequencies of the tobacco occurrences were low (<5%), the ORs from logistic regression approximate PR values; nevertheless, the obtained ORs were corrected using the Miettinen method to estimate PRs^11,12^. This analysis examined the associations between tobacco content and program characteristics across all channels and then separately for children’s and general programming channels. A multivariable logistic regression model, adjusting for potential confounding factors related to program and channel characteristics, examined the associations between tobacco occurrences and specific program subgenres. This model estimated adjusted prevalence ratios (APRs).

In all regression models, the presence or absence of a tobacco occurrence within a one-minute interval served as the dependent variable. The independent variables included the channel and program characteristics broadcast during that minute. Four separate models were run, one for each type of tobacco occurrence. For models with subgenre as an independent variable (unordered categorical), we created k-1 binary (0/1) dummy variables to obtain the PR for each subgenre category.

We originally had 14 subgenre categories; however, creating a dummy for each would have made some categories with sparse data, possibly leading to model instability and unreliable estimates. To reduce the likelihood of these problems and have a meaningful interpretation of the associated subgenres with tobacco content, we combined categories based on *a priori* knowledge or conceptual similarity across subgenres, making sure at the same time that each category included a least 20 minutes of broadcast with tobacco content to favor the stability of the model. The result was the following categories: Daily News, Other News, Series and Feature Films, and chose the ‘Other entertainment’ category as the reference. Besides making sense conceptually, this category was selected as the reference because it had the lowest prevalence of tobacco occurrences, therefore providing for the other categories a PR>1, which facilitated the interpretations of results. We included these k-1 dummy variables as predictors in the logistic binomial model.

Robust variance estimates accounted for the clustering of tobacco occurrences within program units, defined as self-contained content with a defined start and end within the same day.

We established the significance level for PRs at α=0.05. All analyses were conducted using STATA version 13.

To calculate the number of tobacco impressions in each minute containing a tobacco occurrence, we utilized Kantar’s audience measurement data (https://shorturl.at/yCiel), which provided the total number of viewers, and the number of viewers aged 4–24 years watching the specific channel minute by minute. Since these data represent audience size at each minute and do not track individual viewers over time, we could not determine whether the audience composition remained constant or varied from minute to minute. Therefore, to account for this potential audience turnover, we expressed the total number of impressions as person-minutes.

The Ethics Committee of IDIBELL granted an exception to the study since it did not involve human subjects.

## RESULTS

### TV programming

Out of the total 75600 minutes (about 1260 hours) of recorded broadcasting, 318 (0.4%) minutes were lost due to technical difficulties, leaving 75282 minutes (about 1255 hours) available for analysis. After removing 11323 (15.0%) minutes of advertisements and trailers, 63959 minutes (about 1066 hours) of TV programming content were analyzed for tobacco imagery. Analysis revealed that 62.9% of the tobacco content aired on private channels, primarily after 10:00 p.m. (60.2%), with a majority (71.4%) belonging to the entertainment genre and over half (53.9%) being national productions. Mediaset’s private network accounted for 30.8% of the total recorded programming time, representing 48.9% of all programming on private channels.

### Prevalence of tobacco imagery

The content analysis of the total programming time revealed that 2.4% of the programming contained at least one instance of tobacco imagery negative for health. The channels with the highest prevalence of tobacco imagery were FdF (7.0%), owned by Mediaset, and 13TV (5.9%), owned by the Catholic Church. In contrast, channels targeting children, the Disney Channel (owned by Disney Entertainment) and Boing (also owned by Mediaset), had the lowest percentage of tobacco imagery at 0.1% each (see Supplementary file Table S1 for further details).

Further analysis revealed that explicit tobacco use was the most prevalent depiction, accounting for 913 minutes (1.4%) of total programming airtime. Of these, >95% involved combustible tobacco products, while only four instances depicted using a vaporizer device. Additionally, tobacco paraphernalia were observed in 620 minutes, representing 1.0% of the programming time. The presence of tobacco brands was negligible, appearing in less than 0.1% of the total programming time, or 8 minutes (Table 1). Table 2 indicates that out of 1512 minutes depicting tobacco content, about 78% contained only one tobacco occurrence, while the remaining 22% had between 2 and 4 occurrences, with a maximum of five.

**Table 1:** Distribution of program total minutes in prime-time TV by type of tobacco occurrence, Spain, 2021 (N=63959)

*Type of tobacco* *occurrence*	*Number of minutes* *(approximate hours)*	*Percent of overall* *programming*
Explicit	913 (15)	1.4
Implicit	99 (1.7)	0.2
Actual use	957 (16)	1.5
Paraphernalia	620 (10)	1.0
Other	244 (4)	0.4
Cultural cues	807 (13)	1.3
Brand	8 (0.1)	<0.1
At least one	1512 (25)	2.4

**Table 2:** Distribution of programming minutes by number of tobacco occurrences per minute in primetime TV in Spain, 2021

*Number of* *tobacco* *occurrences*	*Minutes* *(approximate* *hours)*	*Percent* *programming* *minutes*	*Percent* *minutes with* *tobacco* *content*
0	62447 (1041)	97.6	0.0
1	1186 (20)	1.9	78.4
2	283 (5)	0.4	18.7
3	40 (0.7)	<0.1	2.6
4	3 (<0.1)	<0.1	0.2
Total	63959 (1066)	100	100

The analyzed broadcast sample revealed a total of 710.7 million person-minutes of any tobacco impressions received across the overall population. This translates to an average of 667000 person-minutes or impressions per hour of prime-time programming broadcast to the entire Spanish TV audience. Similarly, the analyzed sample showed young people aged 4–24 years receiving 8.5 million tobacco impressions of any type, 6.4 million impressions of explicit use, and 2.1 million tobacco impressions of smoke-free violation. This translates to average exposure rates of 8000 impressions of any type, 6000 explicit use impressions, and 2000 smoke-free violation impressions per hour of TV viewing for this age group.

### Tobacco content and program characteristics

Table 3 displays the unadjusted association (prevalence ratio) between tobacco content and several program characteristics: broadcast time, production nationality, program genre and subgenre.

**Table 3:** Association of tobacco occurrences with broadcast time, production nationality, channel ownership, and program genre in prime-time-time TV in Spain, 2021

*Variable*	*Comparison*	*Type of* *occurrence*	*Television channels*					
			*All channels* *(N=63959)*		*Non-children channels* *(N=52796)*		*Children channels* *(N=11163)*	
			PR*	*95% CI*	PR*	*95% CI*	PR*	*95% CI*
**Broadcast time**	During vs after children’s protection schedule	Any type	0.7	0.6–0.8	0.7	0.6–0.8	0.2	0.1–0.4
		Actual use	0.5	0.5–0.6	0.5	0.5–0.6	0.4	0.2–0.9
		Cultural cue	0.7	0.6–0.9	0.8	0.7–0.9	0.1	0–0.3
		SFL violation	0.4	0.3–0.5	0.4	0.3–0.5	0.5	0.2–1.5
**Production nationality**	Produced internationally vs nationally	Any type	1.3	1.2–1.5	2.0	1.8–2.2	0.1	0.04–0.1
		Actual use	1.9	1.7–2.2	2.8	2.5–3.3	0.1	0.08–0.3
		Cultural cue	1.0	0.9–1.1	1.5	1.3–1.8	0.0	0.01–0.06
		SFL violation	2.7	2.1–3.5	3.8	2.9–5.0	0.4	0.2–1.1
**Channel ownership**	Public regional vs Mediaset	Any type	No children channels belonging to public regional governments were analyzed		0.9	0.7–1.0	No children channels belonging to public regional governments were analyzed	
		Actual use			1.1	0.9–1.3		
		Cultural cue			0.7	0.5–0.8		
		SFL violation			0.9	0.6–1.4		
	Public national vs Mediaset	Any type	1.0	0.9–1.2	0.9	0.8–1.1	34.4	8.5–140.1
		Actual use	1.5	1.2–1.8	1.5	1.2–1.8	34.5	4.7–251.1
		Cultural cue	0.8	0.6–1.0	0.6	0.5–0.8	44.8	6.2–323.8
		SFL violation	1.9	1.3–2.6	1.7	1.2–2.4	∞	∞–∞
	Private A3Media vs Mediaset	Any type	No children channels belonging to A3Media were analyzed		1.0	0.8–1.1	No children channels belonging to A3Media were analyzed	
		Actual use			1.1	0.9–1.3		
		Cultural cue			0.9	0.7–1.0		
		SFL violation			0.9	0.6–1.4		
	Private other vs Mediaset	Any type	1.4	1.2–1.7	2.4	2.0–2.8	1.0	0.1–6.9
		Actual use	2.5	2.1–3.0	4.1	3.4–5.0	1.0	0.1–15.5
		Cultural cue	0.6	0.5–0.8	1.0	0.8–1.4	1.0	0.1–15.5
		SFL violation	2.4	1.6–3.4	3.8	2.7–5.5	1.0	1–1
**Broadcast genre**	Entertainment vs news genre	Any type	Children channels do not have news programs		1.8	1.6–2.1	Children channels do not have news programs	
		Actual use			2.0	1.7–2.4		
		Cultural cue			1.9	1.6–2.2		
		SFL violation			7.0	4.4–11.4		

* Prevalence ratio (PR) and 95% confidence interval (CI). SFL: smoke-free legislation.

*Broadcast time*

Tobacco-related content appears significantly more often after the children’s protection schedule ends (1042 instances) than during the scheduled period (470 instances) across all channels and types of tobacco occurrence. There is a 40% greater likelihood of encountering such content after the protection schedule (PR=0.7; 95% CI: 0.6–0.8). Results are similar for depictions of actual tobacco use, cultural cues to tobacco, and smoking where it is banned in Spain. The disparity in the prevalence of tobacco occurrences of any type before and after the protection schedule is considerably more pronounced in channels specifically targeted toward children. In these channels, the prevalence of any type of tobacco occurrence was five times higher after the protection schedule than during it (PR=0.2; 95% CI: 0.1–0.4). Specifically, 74 instances were observed after 10 p.m. compared to11 instances during the children’s protection schedule.

*Production nationality*

Internationally produced content has a 30% higher prevalence of any type of tobacco occurrence (PR=1.3; 95% CI: 1.2–1.5). This pattern is more pronounced for actual tobacco use (PR=1.9; 95% CI: 1.7–2.1) and for non-children TV channels. However, this is not the case for cultural cue occurrences. In the case of channels targeting children, the nationally produced content shows a much higher prevalence of tobacco imagery, seven times higher for actual tobacco use, and 34 times higher for cultural cue occurrences (Table 3).

*Channel ownership*

Compared to the Mediaset channels, the prevalence of actual use occurrences was higher for the national public channels (PR=1.5; 95% CI: 1.2–1.8) and significantly higher for the ‘other’ private channels (PR=4.1; 95% CI: 3.4–5.0). There is no statistically significant difference in the prevalence of actual use occurrences between public regional and A3Media channels and Mediaset channels. The prevalence of cultural cue occurrences was lower on public channels than on Mediaset, while there is no statistically significant difference between the private channels. The prevalence of smoke-free law violation occurrences was also higher for the public national channels (PR=1.7; 95% CI: 1.2–2.4) and the ‘other’ private channels (PR=3.8; 95% CI: 2.7–5.5). Compared to the Mediaset channels, the prevalence of actual tobacco use occurrences is higher for the national public channels.

*Genre*

An analysis of tobacco occurrences by program genre across all TV channels not specifically targeted toward children (excluding children’s channels, which lack news programming) revealed a significantly higher prevalence of any tobacco-related content in entertainment programs compared to news programs (PR=1.8; 95% CI: 1.6–2.1). Furthermore, instances of actual tobacco use were observed 2.0 times more frequently in entertainment programs than in news programs (Table 3). Scenes where smoking happened in places in violation of the smoke-free law were observed seven times more frequently in entertainment programs than in news programs.

*Subgenre*

Table 4 analyzes the associations between TV program subgenres and the prevalence of tobacco imagery content, comparing two distinct models.

**Table 4:** Association between program subgenres and prevalence of tobacco occurrences in general programming channels in prime-time TV in Spain, 2021

*Type of* *tobacco* *occurrence*	*Subgenre*	*Comparison*	*Model A* *unadjusted*		*Model B* *adjusted*	
			*PR*	*95% CI*	APR*	*95% CI*
**Any**	Daily news	Other entertainment	2.2	1.7–2.9	1.8	1.4–2.4
	Other informative		2.2	1.7–2.8	2.1	1.7–2.6
	Films		9.2	7.5–11.3	11.9	9.5–14.9
	Series		3.4	2.8–4.3	4.3	3.4–5.5
**Actual use**	Daily news	Other entertainment	2.3	1.6–3.4	2.0	1.3–3.1
	Other informative		3.5	2.5–4.9	3.5	2.5–4.8
	Films		17.6	13.0–23.8	17.2	12.4–23.8
	Series		3.2	2.3–4.5	3.1	2.2–4.6
**Cultural cue**	Daily news	Other entertainment	2.3	1.6–3.1	1.8	1.2–2.5
	Other informative		1.6	1.1–2.1	1.5	1.1–2.0
	Films		5.5	4.2–7.2	8.6	6.4–11.4
	Series		4.1	3.1–5.3	6.1	4.5–8.3
**SFL violation**	Daily news	Other entertainment	0.6	0.1–5.9	0.3	0.0–3.2
	Other informative		7.9	2.3–26.3	6.9	2.1–23.1
	Films		77.8	24.8–244.1	112.4	34.5–366.1
	Series		25.3	8.0–80.1	39.2	11.9–128.6

* APR: adjusted prevalence ratio; adjusted for time of broadcast, production nationality, and channel ownership. SFL: smoke-free legislation.

Model A examines this association unadjusted for confounding factors. The results indicate that feature films present the highest prevalence of tobacco content compared with all other program subgenres. Specifically, the prevalence of any tobacco imagery in feature films is estimated to be 9.2 times higher than that in other entertainment programs (95% CI: 7.5–11.3). Series also demonstrated a heightened prevalence, with any tobacco imagery appearing approximately three times more often than in other entertainment programs (PR=3.4; 95% CI: 2.8–4.3). While less pronounced than in feature films and series, other subgenres, such as daily news and informative programs, also display a higher prevalence of tobacco imagery than other entertainment programs do (PR=2.2; 95% CI: 1.7–2.9).

Furthermore, Model A reveals that specific types of tobacco depictions are significantly more common in feature films. Compared with other entertainment programs, feature films depict the actual use of tobacco, the presence of cultural cues related to tobacco, and smoking in locations prohibited by Spanish law nearly 18, 6, and 80 times more often, respectively. These same types of tobacco depictions are also more prevalent in series, occurring approximately 3, 4, and 25 times more often than in other entertainment programs.

Model B investigates the same associations after adjusting for the time of broadcast, production nationality, and channel ownership as potential confounders. This adjustment maintains the statistical significance observed in Model A; however, it notably strengthens the association between feature films and each type of tobacco occurrence. Compared with other entertainment programs, feature films depict any tobacco occurrence, actual tobacco use, cultural cues related to tobacco, and smoking in prohibited places 12, 17, 9, and 112 times more often (Table 4), respectively. Series also exhibit elevated frequencies of these depictions, appearing roughly 4, 3, 6, and 39 times more often than in other entertainment programs. Further analysis (not shown) indicates that production nationality is the primary confounding factor. Non-Spanish feature films were twice as likely to have been produced before 2011, when Spain implemented comprehensive tobacco control legislation. Some PR estimates showed wide CIs due to small sample sizes for some cells and even sparse data or zero cell counts.

Finally, the analysis of children’s channels revealed very high associations for feature films and series because of quasi-separation in the data and sparse data bias. Consequently, these PR values are not reported for children’s channels, as they lack practical interpretability and may be misleading.

## DISCUSSION

This analysis of Spanish TV programming reveals that tobacco imagery occurred during 2.4% of airtime in 2021, with actual tobacco use being the most prevalent depiction. This prevalence is slightly lower than that reported in similar studies in Chile (2.7% in 2019^13^) and the UK (2.9% in 2010^8^ and 2.8% in 2015^14^). However, the proportion of airtime featuring explicit tobacco use (1.4%) is consistent with that reported in the Chilean study but higher than that observed in the UK studies, where such depictions occurred at 1% one-minute intervals. Despite the lower overall prevalence of tobacco imagery, this study identified a slightly greater proportion of airtime depicting smoking in areas prohibited by Spanish smoke-free legislation. Finally, the presence of tobacco brands in Spanish programming was minimal (0.01%), notably lower than in both the Chilean study (0.15%) and the 2010 UK study (0.1%). This finding, however, aligns with the 2015 UK study, which also reported a very low prevalence of tobacco branding (0.04%).

The 2.4% prevalence of tobacco imagery on Spanish TV should not be underestimated. Even this apparently modest content level of tobacco imagery translates into a substantial number of impressions for young viewers aged 4–24 years: 8.5 million impressions of any type, 6.5 million depicting tobacco use, and 2.1 million portraying violations of smoke-free policies. These correspond to rates of 8000, 6000, and 2000 impressions per hour of TV viewing, respectively, for this age group. A similar study conducted in Chile^9^ found that 2.7% of minutes with tobacco content delivered seemingly higher tobacco impression rates for the age group of 4–24 years: 21800 for any type, 8000 for tobacco use, and 2100 for smoke-free violations per hour of viewing. These observed differences may be attributed to variations in both tobacco content TV programming and audience sizes and behaviors between the two countries.

These findings indicate that even a seemingly low prevalence of tobacco content in TV programming can result in substantial population-level exposure to tobacco imagery, with a particularly pronounced impact on youth. This pervasive exposure to tobacco imagery can influence viewers’ perceptions of tobacco use, contributing to its normalization and potentially increasing the risk of initiation and experimentation.

While the prevalence of tobacco-related content on Spanish TV was lower during the designated children’s protection schedule than afterward, its presence remains a significant concern. Even within these protected hours, 470 instances of tobacco imagery were identified, resulting in an estimated 15.6 million tobacco impressions. This finding indicates that children are not fully shielded from such content and underscores the need for further protective measures. Moreover, it is important to acknowledge that many young people continue to watch TV beyond the designated protection schedule, exposing them to a significantly higher prevalence of tobacco occurrences (1042 instances). In 2018, children’s TV consumption in Spain peaked outside legally protected hours^15^. During prime time (10:00 p.m. to midnight), over 1.2 million viewers aged <18 years watched TV, representing 19% of that age group. Even more concerning, 356000 children (5.4% of minors) remain glued to the screen past midnight. This highlights the inadequacy of current child protection measures and the need for stricter regulations and parental supervision to protect children from potentially harmful content.

Feature films stand out as a significant source of tobacco occurrences on TV, especially in depictions of violations of Spain’s smoke-free legislation and actual tobacco use. Compared to other entertainment programs, feature films were 112 times more likely to show scenes with violations of the smoke-free law and 17 times more likely to portray actual tobacco use, even after accounting for factors such as broadcast time, channel ownership, and production nationality. This pattern also appears to extend to children’s channels, although the specific PRs for these channels should be interpreted with caution due to potential biases.

Numerous studies have demonstrated a strong association between exposure to smoking in films and the likelihood of adolescents initiating smoking. This finding is consistent across both cross-sectional and longitudinal research in the United States and Europe^16-19^. This association persists even after controlling for potential confounders such as socio-economic status, parental smoking, and peer influence. Furthermore, the effect appears to be dose-dependent, meaning greater exposure to smoking in films corresponds to a greater risk of smoking initiation^17^. Some studies even suggest that movie smoking could contribute to as much as one-third of smoking initiation among young adolescents^18^ and that exposure to tobacco imagery in episodic programs on streaming platforms and TV is also common and may impact future e-cigarette use^20,21^. Therefore, reducing tobacco imagery in films and series represents a promising strategy for curbing youth smoking^22,23^.

While the impact of TV broadcast films on smoking initiation is likely similar to that shown on the big screen, it is less studied. Research consistently indicates a significant relationship between TV viewing in general and smoking behaviors. Increased TV viewing is associated not only with a greater likelihood of young people starting to smoke, but also an earlier smoking initiation in adolescents^24,25^. Moreover, TV viewing may increase the likelihood that young adult women intend to smoke and decrease their ability to refuse cigarettes^26^. It also shapes adolescents’ perceptions of smoking prevalence and the image of smokers^27^. Given the prevalence of tobacco imagery in films and episodic programs on TV and streaming platforms, it is crucial to consider the potential influence of this exposure on future smoking and e-cigarette use among youth.

This study’s findings on tobacco imagery in Spanish television have global implications. The prevalence of tobacco imagery on television, particularly in feature films, is a global concern^28^. This research highlights the influence of international productions, the vulnerability of children, and the need for stronger tobacco control measures, encouraging similar research and policy action in other countries.

### Strengths and limitations

Our study has certain limitations. The study was conducted during the COVID-19 pandemic, which may have influenced TV programming and viewership. We also controlled for key variables, but the potential for residual confounding from unmeasured factors remains. While our sample is susceptible to seasonal variations in content; this likely did not affect our findings since the primary sources of tobacco imagery were consistently aired programs. The coding methodology itself carries potential limitations: we included any tobacco or nicotine product, including vaping, in the explicit use category without the ability to analyze specific products like e-cigarettes, and there is always a possibility of classification bias in interpreting and categorizing visual content despite coder training and reliability checks. We coded only non-pay channels with the highest viewing share. We should be aware that young people may access other platforms, such as VOD platforms, but the lack of public viewership data for VOD hinders the inclusion of these programs in our study. Finally, generalizing these findings to other countries with different media landscapes and regulations should be done with caution.

A key strength of our study is its large sample size of recorded TV programming minutes, enabling a comprehensive assessment of tobacco imagery across a wide range of TV programming. Additionally, despite being time-intensive, our approach of coding one-minute intervals allowed for high granularity and accuracy in data collection. This study is the first in Spain and second in Europe to provide a population estimate of the number of tobacco impressions over time from watching overall general TV programming. Replication of these cross-sectional results in other European countries would contribute to a better understanding of the impact of tobacco imagery and implications for tobacco control. Moreover, repeated cross-sectional studies in the same populations would contribute to monitoring control measures addressing tobacco imagery.

## CONCLUSIONS

This study highlights an urgent need to better protect young people from tobacco imagery on TV. The current reliance on youth protection schedules is inadequate. Spanish authorities should implement key recommendations from the WHO FCTC Article 13 Implementation Guidelines^29^. These include mandating strong anti-tobacco disclaimers at the beginning of any program containing tobacco imagery and requiring ‘no-pay-off’ certifications to ensure that no financial incentives are involved in depictions of tobacco. Furthermore, media productions containing tobacco imagery should be ineligible for public subsidies^30^. Policies supporting media production, whether for cultural or commercial purposes, must align with the fundamental public health obligation to protect citizens from tobacco promotion. By adopting these measures, Spain can better safeguard the health of future generations and fulfill its commitments under the WHO FCTC.

## Supplementary Material



## Data Availability

The data supporting this research are available from the authors on reasonable request.
