# Supplementary material for: Tobacco imagery in prime-time television in Spain: A content analysis
Source: Tob Induc Dis. 2025 Jun 10;23:10.18332/tid/204750. doi: 10.18332/tid/204750 (PMC12150290; doi:10.18332/tid/204750)
Supplement: Supplementary file 1 [file TID-23-79-s1.pdf]

TABLE S 1

MINUTES OF TV BROADCAST BY TYPE OF CHANNEL, PROGRAM GENRE, PRODUCTION NATIONALITY AND TYPE OF TOBACCO OCCURRENCE

in prime- time TV in Spain, 2021

			MINUTES OF					GENRE			PRODUCTION NATIONALITY				TYPE OF OCCURRENCE			
OWNERSHIP	CORPORATION	CHANNEL	Regular Programming	Commercial break	Missing	Total	% missing	Entertainment	News	% entertainment	Spanish	USA	Other	% national production	Any	% of programming	Tobacco use	% of programming
Private	A3media	A3	3,358	836	6	4,200	0.1%	2,755	603	82.0%	2,327	279	752	69.3%	51	1.5%	28	0.8%
Private	A3media	La6	3,305	895	0	4,200	0.0%	1,392	1,913	42.1%	2,732	475	98	82.7%	101	3.1%	45	1.4%
Private	A3media	Neox	3,313	887	0	4,200	0.0%	3,313	0	100.0%	95	2,915	303	2.9%	144	4.3%	101	3.0%
Private	A3media	Nova	3,343	853	4	4,200	0.1%	3,295	48	98.6%	48	653	2,642	1.4%	39	1.2%	15	0.4%
Private	Mediaset	4TV	3,184	951	65	4,200	1.5%	1,786	1,398	56.1%	2,768	313	103	86.9%	46	1.4%	17	0.5%
Private	Mediaset	Boing	3,458	704	38	4,200	0.9%	3,458	0	100.0%	0	2,875	583	0.0%	2	0.1%	1	0.0%
Private	Mediaset	Divinity	3,245	901	54	4,200	1.3%	3,245	0	100.0%	400	452	2,393	12.3%	36	1.1%	19	0.6%
Private	Mediaset	Energy	3,253	933	14	4,200	0.3%	3,253	0	100.0%	0	3,253	0	0.0%	40	1.2%	27	0.8%
Private	Mediaset	FdF	3,268	932	0	4,200	0.0%	3,268	0	100.0%	2,060	1,033	175	63.0%	228	7.0%	117	3.6%
Private	Mediaset	T5	3,258	887	55	4,200	1.3%	2,466	792	75.7%	3,023	141	94	92.8%	70	2.1%	56	1.7%
Private	Other	13TV	3,713	423	64	4,200	1.5%	1,202	2,511	32.4%	2,657	822	234	71.6%	220	5.9%	194	5.2%
Private	Other	Disney	3,558	642	0	4,200	0.0%	3,558	0	100.0%	679	2,669	210	19.1%	2	0.1%	1	0.0%
Total Private			40,256	9,844	300	50,400	0.6%	32,991	7,265	82.0%	16,789	15,880	7,587	41.7%	979	2.4%	621	1.5%
Public	National	Clan	4,147	49	4	4,200	0.1%	4,143	0	99.9%	992	2,965	190	23.9%	81	2.0%	41	1.0%
Public	National	La1	4,076	119	5	4,200	0.1%	2,202	1,874	54.0%	2,966	982	128	72.8%	128	3.1%	102	2.5%
Public	National	La2	4,072	128	0	4,200	0.0%	1,445	2,627	35.5%	2,848	233	991	69.9%	67	1.6%	55	1.4%
Public	Regional	ETB1	3,957	240	3	4,200	0.1%	1,622	2,335	41.0%	3,795	0	162	95.9%	31	0.8%	5	0.1%
Public	Regional	TV3	3,545	650	5	4,200	0.1%	1,164	2,381	32.8%	3,194	129	222	90.1%	139	3.9%	110	3.1%
Public	Regional	TVG	3,906	293	1	4,200	0.0%	2,118	1,788	54.2%	3,906	0	0	100.0%	87	2.2%	23	0.6%
Total public			23,703	1,479	18	25,200	0.1%	12,694	11,005	53.6%	17,701	4309	1693	74.7%	533	2.2%	336	1.4%
		Total	63,959	11,323	318	75,600	0.7%	45,685	18,270	71.4%	34,490	20,189	9,280	53.9%	1,512	2.4%	957	1.5%

Table S2

Minutes by type of tobacco occurrence

in prime- time TV in Spain, 2021

	Any	Tobacco use	Other	SFL Violation	Tobacco brand	Total minutes
News	129	58	82	1	0	7,001
Other News	207	144	93	21	1	11,484
Feature Film	630	523	248	157	1	8,903
Series	355	143	263	74	4	12,636
Other Entertainment	106	46	66	3	2	12,772

© 2025 Peruga A. et al.
